# Targeting Sphingolipids in Breast Cancer: From Tumor Biology to Therapeutic Strategies

**DOI:** 10.32604/or.2025.071523

**Published:** 2026-01-19

**Authors:** Min Hee Kim, Boyoon Huh, Joo-Won Park, Woo-Jae Park

**Affiliations:** 1 Department of Biochemistry, College of Medicine, Ewha Womans University, Seoul, 07804, Republic of Korea; 2 Department of Biochemistry, Chung-Ang University College of Medicine, Seoul, 06974, Republic of Korea

**Keywords:** breast cancer, sphingolipid, drug resistance, metastasis, metabolism

## Abstract

Breast cancer is one of the most prevalent malignancies among women and comprises a heterogeneous spectrum of molecular subtypes with distinct biological behaviors. Among various regulatory molecules, sphingolipids play pivotal roles in dynamically modulating fundamental cellular processes such as proliferation, apoptosis, and metastasis through metabolic interconversions, including phosphorylation, glycosylation, and the generation of sphingosine-1-phosphate. This review aims to elucidate the mechanisms through which sphingolipid metabolism orchestrates cancer cell fate and drives breast cancer progression. Particular emphasis is placed on the balance between proapoptotic ceramides and pro-survival metabolites, such as sphingosine-1-phosphate, which collectively influence tumor growth and the therapeutic response. Additional sphingolipid species, including glucosylceramide and gangliosides (GD2, GD3, GM1, and GM3), have also been implicated in promoting breast cancer development. Furthermore, sphingolipid-based therapeutic strategies, including immunotherapy and antibody therapy, are discussed. By providing a comprehensive overview of sphingolipid metabolism, this review aims to identify novel therapeutic targets that may help overcome treatment resistance and improve clinical outcomes in breast cancer.

## 1 Introduction

Breast cancer represents the most common cancer in women and, coincidentally, the second leading cause of cancer death among women. Various signaling pathways, including the estrogen receptor (ER), progesterone receptor (PR), epidermal growth factor (EGF), receptor tyrosine-protein kinase erbB-2 (HER2), and vascular endothelial growth factor (VEGF), are involved in the pathophysiology of breast cancer; meanwhile, breast cancer subtypes can be classified according to the expression of ER, PR, and HER2. Indeed, the four major molecular subtypes include luminal A (ER+, PR+/−, HER2−, Ki-67 low), luminal B (ER+, PR+/−, HER2+/−, Ki-67 high), HER2 positive (ER−, PR−, HER2+), and triple-negative (ER−, PR−, HER2−) breast cancer (TNBC) [1]. Among these, TNBC presents significant therapeutic challenges due to the lack of targetable receptors and is associated with aggressive tumor behavior, high metastatic potential, and poor prognosis.

Sphingolipids (SLs) have emerged as central regulators of various cellular processes since the “SL rheostat” was announced 25 years ago, which refers to the balance between proapoptotic ceramide and pro-survival sphingosine-1-phosphate (S1P) that determines the cell fate [2]. Dysregulated SL metabolism plays a crucial role in determining tumor cell behavior in breast cancer, including cancer proliferation, survival, apoptosis and metastasis [3]. Moreover, accumulating evidence highlights that alterations in SL signaling are not merely secondary phenomena but actively contribute to breast cancer progression and therapeutic resistance. Furthermore, numerous anticancer reagents, including doxorubicin, tamoxifen, and paclitaxel, display additional effects in modulating SL metabolism [3,4]. Thus, targeting SL-related pathways has emerged as a promising therapeutic strategy for breast cancer. Despite the identification of diverse SL species, the functional roles of these species remain incompletely characterized. SLs display remarkable structural heterogeneity determined by fatty acyl chain length, sphingoid base variants (e.g., sphingosine, sphinganine), and glycosylation complexity, as well as by bioactive derivatives such as S1P and ceramide-1-phosphate (C1P) [3].

This review summarizes recent advances in understanding how SL metabolic pathways regulate breast cancer progression and cell death mechanisms, and discusses the therapeutic potential of short- and medium-chain ceramides (C2–C8) as novel anticancer agents, with emphasis on translational opportunities and current challenges. Additionally, this review addresses the emerging roles of SLs in immunotherapy and antibody-based therapeutic strategies. The aim of this review is to integrate recent advances in SL biology and SL-based therapeutics to delineate novel strategies that can improve clinical outcomes in breast cancer.

## 2 Background

**Figure 1:**
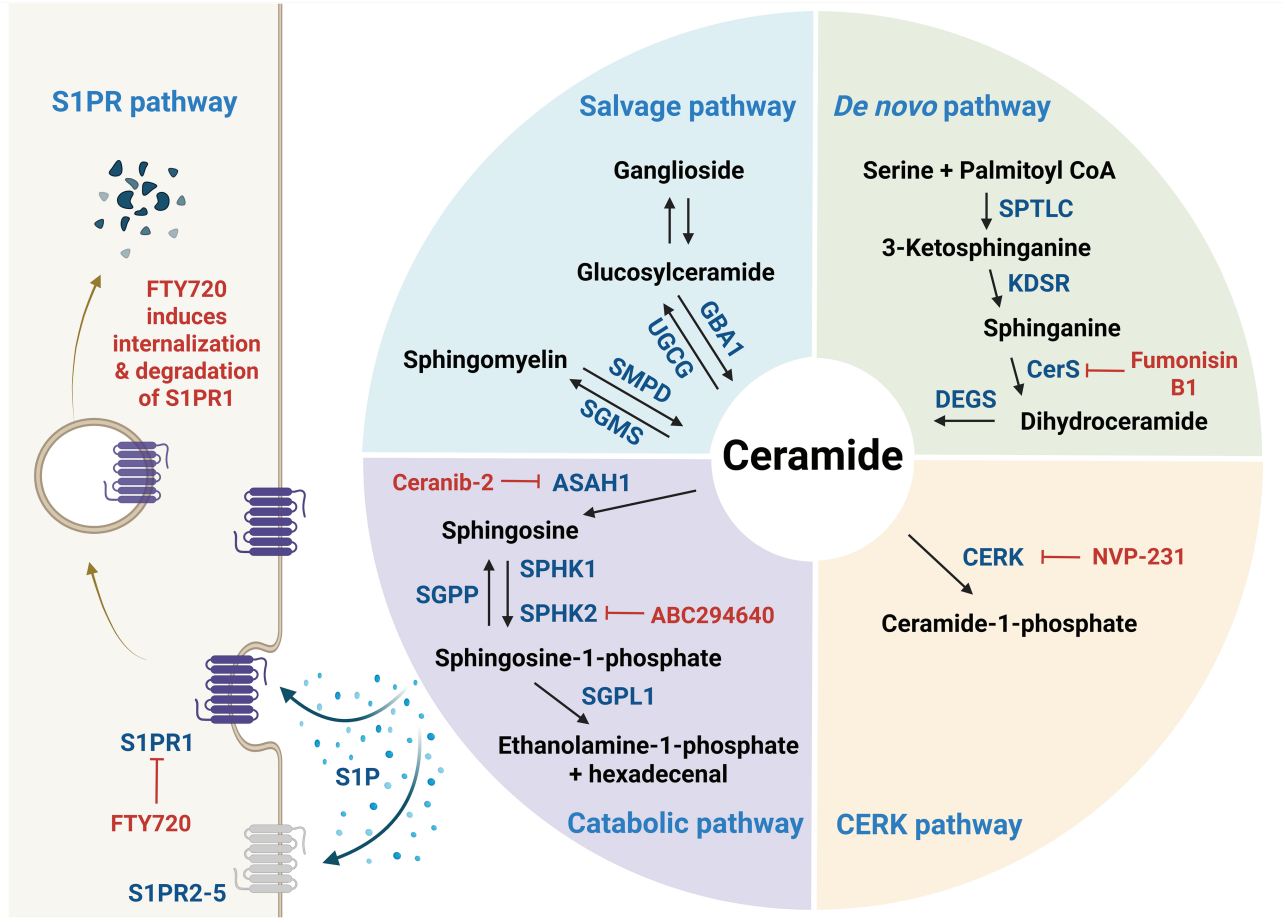
Sphingolipid metabolism. *De novo* ceramide synthesis begins with the condensation of serine and palmitoyl-CoA catalyzed by serine palmitoyltransferase (SPTLC), the rate-limiting enzyme in the pathway, which produces 3-ketosphinganine. This intermediate is subsequently reduced by 3-ketosphinganine reductase (KDSR) to form sphinganine, which then undergoes N-acylation by ceramide synthase (CerS) with fatty acyl-CoAs of varying chain lengths to yield dihydroceramide. Next, dihydroceramide desaturase (DEGS) introduces a trans double bond to form ceramide, the central hub in sphingolipid metabolism. Ceramide can be converted into diverse sphingolipid species: ceramide-1-phosphate (C1P) via ceramide kinase (CERK), sphingomyelin via sphingomyelin synthase (SGMS), and glucosylceramide via glucosylceramide synthase (UGCG), which can be further modified into complex gangliosides such as GM3, GD3, and GT3 through the sequential addition of sugar residues and N-acetylneuraminic acid. Ceramide is degraded by ceramidase (CDase, encoded by *ASAH1* (*N-acylsphingosine amidohydrolase*) gene) to sphingosine, which is then phosphorylated by sphingosine kinase (SphK) to form sphingosine-1-phosphate (S1P), a bioactive lipid involved in various cellular signaling processes. S1P, depicted as blue dots surrounding the arrows, can be exported from cells to act in either an autocrine or paracrine manner through its G protein-coupled receptors (S1PR1–5); FTY720 functionally inhibits S1PR1. The figure was created in BioRender.com, accessed on 03 July 2025. Abbreviation: ASAH1, N-acylsphingosine amidohydrolase; CERK, ceramide kinase; CerS, ceramide synthase; DEGS, dihydroceramide desaturase; GBA1, glucosylceramidase beta 1; KDSR, 3-ketosphinganine reductase; SGMS, sphingomyelin synthase; SGPL1, sphingosine-1-phospohate lyase 1; SGPP, sphingosine-1-phosphate phosphatase; SMPD, sphingomyelin phosphodiesterase; SPHK, sphingosine kinase; SPTLC, serine palmitoyltransferase; S1P, sphingosine-1-phosphate; S1PR, S1P receptor; UGCG, glucosylceramide synthase

**Figure 2:**
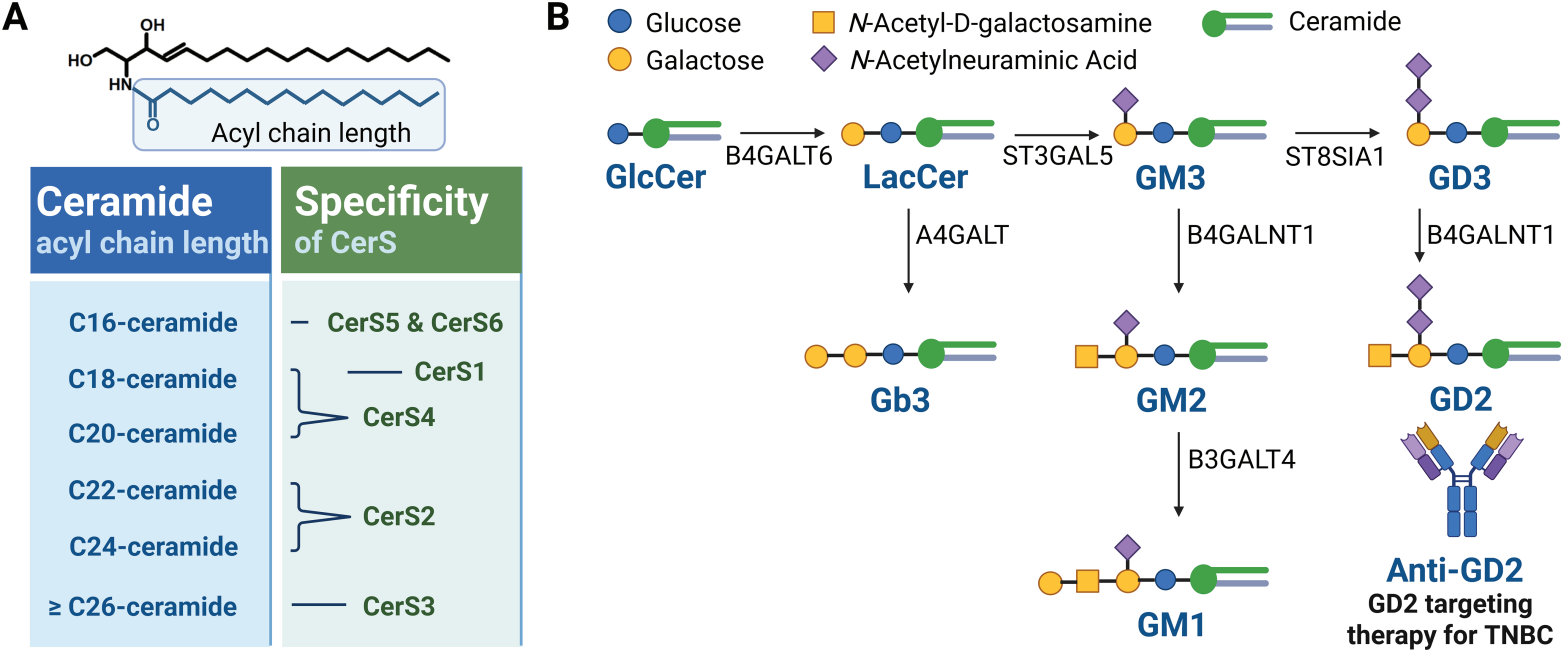
Characteristics of CerS and the glycosphingolipid metabolic pathway. (**A**) Six mammalian isoforms of ceramide synthases (CerS1–CerS6) catalyze the N-acylation of sphingoid bases using fatty acyl-CoAs of defined chain lengths to generate ceramides with distinct acyl chain compositions. CerS1 preferentially utilizes C18 fatty acyl-CoA to produce C18-ceramide. CerS2 predominantly synthesizes very long-chain ceramides such as C22–C24-ceramides. CerS3 is responsible for generating ultra-long-chain ceramides (≥C26), especially important in skin barrier function. CerS4 favors C18–C20 fatty acyl-CoAs, producing C18–C20-ceramides. CerS5 and CerS6 both preferentially incorporate C16 fatty acyl-CoA, leading to the production of C16-ceramide, which is commonly implicated in apoptotic signaling. (**B**) Ceramides serve as central intermediates in the glycosphingolipid biosynthesis pathway. Ceramide is converted to glucosylceramide (GlcCer) via GCS-mediated glycosylation. GlcCer serves as a precursor for more complex glycosphingolipids, including lactosylceramide (LacCer), gangliosides such as GM3 and GD3, and globosides such as Gb3. A representative schematic of the glycosphingolipid metabolic pathway implicated in breast cancer development is presented. The figure was created with BioRender.com, accessed on 03 July 2025. Abbreviation: A4GALT, alpha 1,4-galactosyltransferase; B3GALT4, beta-1,3-galactosyltransferase 4; B4GALT1, beta-1,4-galactosyltransferase 1; B4GALT6, beta-1,4-galactosyltransferase 6; GCS, glucosylceramide synthase; GlcCer, glucosylceramide; LacCer, lactosylceramide

*De novo* SL biosynthesis (Fig. 1) is initiated by the enzyme serine palmitoyltransferase (SPTLC1–3), which catalyzes serine and palmitoyl-CoA condensation to form 3-ketosphinganine; this reaction represents the rate-limiting step in the *de novo* synthesis of ceramide [3]. Meanwhile, 3-ketosphinganine reductase (KDSR) catalyzes the reduction of 3-ketosphinganine to sphinganine, which is subsequently acylated by ceramide synthases (CerS) through the attachment of fatty acyl-CoA, yielding dihydroceramide. Then, dihydroceramide is converted to ceramide via desaturation by dihydroceramide desaturase (DEGS) [3]. Among the enzymes involved, six isoforms of CerS exhibit substrate specificity for fatty acyl-CoA of distinct chain lengths, thereby generating ceramides in mammals with varying acyl chain lengths [3]. For instance, CerS1 preferentially produces C18-ceramide [5], while CerS2 synthesizes C22–C24 ceramides [6]. Additionally, CerS3 is responsible for producing ceramides with acyl chains longer than C26 [7], while CerS4 primarily generates C18–C20 ceramides [8]. CerS5 and CerS6 both produce C16-ceramide (Fig. 2A) [9,10].

Ceramide serves as a central hub in SL metabolism and can be further metabolized into numerous bioactive SLs, including C1P by ceramide kinase (CERK), glucosylceramide by glucosylceramide synthase (UGCG or GCS), and sphingomyelin by sphingomyelin synthase (SGMS) (Fig. 1) [3]. Sphingomyelin serves as a major reservoir for ceramide production and is particularly enriched in cellular membranes. The hydrolysis of sphingomyelin into ceramide is catalyzed by sphingomyelinases (SMases), which are primarily located in the plasma membrane and lysosomes. Specifically, neutral SMase is predominantly found at the plasma membrane, while acid SMase is localized within lysosomes. There are four characterized types of SMases (*SMPD1–SMPD4*), each of which exhibits distinct enzymatic properties and contributes differently to the pathogenesis of various diseases. Glucosylceramide can be further processed to lactosylceramide, which serves as a precursor for thousands of complex glycosphingolipids such as gangliosides containing sialic acids (e.g., GM1–3, GD1–3, and GT1–3) (Fig. 2B) [11].

Ceramide can also be catabolized into sphingosine by ceramidase (CDase or ASAH). Sphingosine is phosphorylated by sphingosine kinases (SphKs), particularly SphK1 and SphK2, to produce S1P (Fig. 1). SphK1 is predominantly localized in the cytoplasm and migrates to the plasma membrane upon activation. In contrast, SphK2 resides mainly in the nucleus, mitochondria, and endoplasmic reticulum, which promotes the spatially distinct generation of S1P [12]. Meanwhile, S1P produced by SphK1 can be exported from the cell and acts extracellularly by binding to a family of five specific G protein-coupled receptors, known as the S1P receptors (S1PR1–5), each of which mediates distinct cellular responses. Consequently, the biological effects of S1P are highly dependent on the subcellular site of its production and the receptor subtype with which it engages. S1P signaling is tightly regulated by its degradation through two major pathways: dephosphorylation by S1P phosphatases (SGPPs) and irreversible cleavage by S1P lyase.

### 2.1 Mechanisms of Ceramide-Induced Cytotoxicity in Breast Cancer Cells

**Figure 3:**
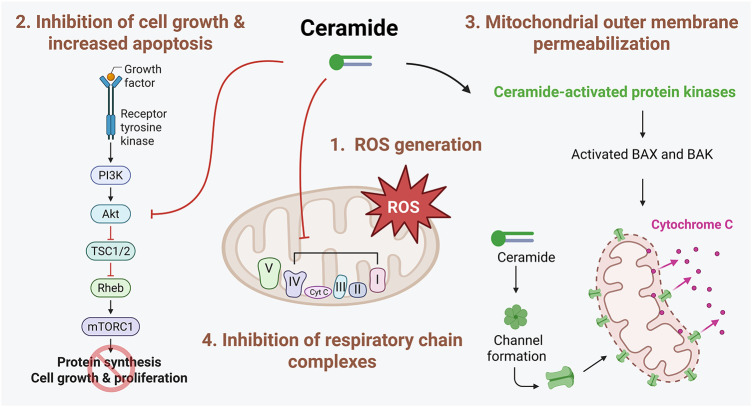
Mechanisms involved in ceramide-induced cell death. Ceramide promotes cell death through multiple interconnected pathways. First, ceramide disrupts mitochondrial function by inhibiting components in the mitochondrial respiratory chain, leading to increased generation of reactive oxygen species (ROS). This oxidative stress contributes to mitochondrial dysfunction and apoptosis. Second, ceramide negatively regulates key pro-survival signaling pathways, including the Akt pathway, thereby reducing cellular proliferation and survival. Additionally, ceramide can directly form channels in the mitochondrial outer membrane or indirectly activate proapoptotic Bcl-2 family proteins, such as BCL2 Associated X, Apoptosis Regulator (BAX) and BCL2 antagonist/killer (BAK). This activation facilitates mitochondrial outer membrane permeabilization (MOMP), resulting in the release of cytochrome c and subsequent activation of the intrinsic apoptotic cascade. The figure was created with BioRender.com, accessed on 03 July 2025. Abbreviation: Akt, protein kinase B; BAX, BCL2 Associated X, Apoptosis Regulator; BAK, BCL2 antagonist/killer; mTORC1, mammalian target of rapamycin complex 1; PI3K, phosphatidylinositol 3-kinase; Rheb, Ras homolog enriched in brain; ROS, reactive oxygen species; TSC1/2, tuberous sclerosis complex 1/2

The SL rheostat refers to a conceptual model describing the dynamic balance between two key bioactive SLs, ceramide and S1P, which determines cell fate [2]. According to the model, ceramide promotes cell cycle arrest and apoptosis, while S1P induces cell survival and proliferation. Therefore, increased ceramide in breast cancer would lead to cell death; several drugs that are employed for breast cancer are known to increase ceramide synthesis by either activating *de novo* ceramide synthesis or sphingomyelin catabolism. For example, doxorubicin primarily elevates C16-ceramide [13], while paclitaxel induces a dose-dependent elevation of total ceramide in MDA-MB-468 and MCF-7 cells [14]. Fenretinide similarly enhances C18- and C24-ceramide in MCF-7 cells [15]. Especially, C16-ceramide acts as a key mediator of cell death induced by chemotherapy, radiation, and inflammation through modulation of endoplasmic reticulum stress [16] and mitochondrial function [17]. Similar to C16-ceramide, novel ceramide analogs and short-chain ceramides (e.g., C2- or C6-ceramide) have efficiently reduced breast cancer cell growth [18–20], supporting the therapeutic potential of strategies that promote intracellular ceramide accumulation. The following mechanisms of ceramide-induced cytotoxicity have been proposed (Fig. 3).

#### 2.1.1 Reactive Oxygen Species (ROS) Generation

Exposure of cell-permeable C6-ceramide caused apoptosis in MDA-MB-435 breast cancer cells, which was potentiated by the concomitant treatment of either a CDase inhibitor or a GCS inhibitor [21]. This phenomenon was associated with increased ROS generation. C6-ceramide-induced apoptosis was inhibited by antioxidants, N-acetylcysteine and glutathione, but not by the pan-caspase inhibitor z-VAD-fmk [21]. These findings indicate that apoptosis caused by intracellular ceramide accumulation is mediated via ROS production [21]. Similarly, the overexpression of glutathione peroxidase-1, the cytosolic thiol peroxidase responsible for peroxide detoxification, reduced doxorubicin-induced ceramide accumulation and subsequent apoptotic cell death in T47D human breast cancer cells [22]. Collectively, these findings highlight the pivotal role of ROS in mediating ceramide-induced cell death in breast cancer.

#### 2.1.2 Induction of Apoptosis and Concomitant Inhibition of Cell Growth

In accordance with the ‘SL rheostat’, ceramide has been nominated to induce cell death and inhibit cell proliferation; meanwhile, several downstream signaling targets, such as ceramide activated protein kinases (CAPKs), protein phosphatase (PP) 1, PP2A, and cathepsin D, have also been identified [23]. These CAPKs include the kinase suppressor of RAS (KSR) and the ζ isotype of protein kinase C (PKCζ) [23]. Ceramide can directly bind to and activate PKCζ, which sequentially promotes apoptosis by regulating nuclear factor-κB (NF-κB) cascades and the formation of proapoptotic complexes with prostate apoptosis response-4 (PAR-4) [24]. In addition, ceramide directly interacts with and activates cathepsin D, which is linked to the cleavage of various proteins, including Bid, subsequently activating the mitochondrial apoptotic pathway [24]. Ceramide also leads to cell cycle arrest via PP1 activation, which dephosphorylates retinoblastoma protein (Rb) [25]. Furthermore, ceramide-activated PP2A inhibits the protein kinase B (AKT)/mammalian target of rapamycin (mTOR) signaling pathway, inhibiting cell growth. Finally, we also reported that C16-ceramide derived from CerS6 inhibits the proliferation of MCF-7 cells by suppressing the mTOR signaling [26].

#### 2.1.3 Formation of Ceramide-Induced Channel and Ceramide-Enriched Domains

Two key proapoptotic members of the B-cell lymphoma 2 (Bcl-2) family, Bcl-2-associated X protein (BAX) and Bcl-2 homologous antagonist/killer (BAK), play a critical role in apoptosis, specifically regulating mitochondrial outer membrane permeabilization (MOMP). Ceramide can directly induce channel formation in membranes, leading to MOMP [27] and cooperate with BAX and BAK to modulate this function [28]. C2- or C16-ceramide primarily induces ceramide channel formation in mitochondria [27], but these are individually disturbed by very long-chain ceramides, such as C24-ceramide [17]. MOMP induced by ceramide channel formation is not restricted to cytochrome c, and other proteins, such as adenylate kinase, were also released from mitochondria [27]. Interestingly, antiapoptotic Bcl-2 family proteins, including Bcl-xL, disassembled ceramide channels, preventing ceramide-induced apoptosis [29]. In addition to MOMP, ceramide can also modulate apoptosis via ceramide-enriched membrane domains [30]. Various stimuli, such as CD95, CD40, tumor necrosis factor-α, irradiation, and cisplatin, have been shown to accelerate the formation of ceramide-enriched domains, triggering apoptosis [30].

#### 2.1.4 Inhibition of Mitochondrial Respiratory Chain Function

Direct inhibition of mitochondrial respiratory chain complexes by ceramide and the subsequent generation of ROS are well-established phenomena. Specifically, C2-ceramide has been shown to impair the functions of complexes I, III, and IV in the respiratory chain, while C16-ceramide selectively inhibits complex IV activity [31–33]. This disruption in mitochondrial respiratory function is associated with increased ROS production, as observed in CerS2-null mice [31], in which the deficiency of very long-chain ceramides leads to elevated levels of C16-ceramide [31]. This disruption is also accompanied by a loss of mitochondrial membrane potential and a marked depletion of intracellular ATP [34]. Recently, ceramide was reported to bind directly to LC3-II, suggesting a novel mechanism through which ceramide regulates autophagy. Accumulation of ceramide in mitochondria was shown to trigger mitophagy through the recruitment of autophagosomes, ultimately leading to mitochondrial degradation [35]. These findings indicate that ceramide may exert dual roles in mitochondrial function, exerting both detrimental and protective effects. While ceramide is known to promote damaging effects, such as stimulating mitochondrial dysfunction and cell death, it also contributes to mitochondrial quality control by facilitating the selective removal of damaged mitochondria through mitophagy, thereby maintaining cellular homeostasis.

### 2.2 SLs as Modulators of Drug Resistance in Breast Cancer

Drug resistance constitutes a critical impediment in breast cancer, markedly undermining the therapeutic efficacy of chemotherapy. Recently, the mechanistic involvement of SLs in the development of chemoresistance has been reported, consequently contributing to the progression and metastasis of breast cancer. Several factors can contribute to SL-induced drug resistance.

#### 2.2.1 ATP-Binding Cassette (ABC) Transporters

ABC transporters utilize ATP hydrolysis to promote the active transport of a wide range of substrates across cellular membranes. Notably, these transporters contribute significantly to chemoresistance in breast cancer by mediating the efflux of therapeutic agents from cancer cells. Indeed, elevated glucosylceramide levels have been observed in tumors from chemotherapy-resistant patients [36]. Inhibiting GCS, the enzyme responsible for ceramide glycosylation, has been shown to prevent MDR1 (*ABCB1*) upregulation in MDA-MB-435 breast cancer cells [37]. Beyond the role of glucosylceramide, stable overexpression of CerS4 in MCF-7 cells led to increased expression of several ABC transporter genes, including *ABCB1* (*MDR1*), *ABCC1* (*MRP1*), *ABCC2* (*MRP2*), *ABCC4* (*MRP4*), and *ABCG2* (*breast cancer resistance protein*), which are associated with the acquisition of chemoresistance [38]. Notably, *ABCA12* was recently identified as a ceramide transporter that exports ceramide from cells, thereby decreasing intracellular ceramide levels [39]. Given the role of ceramide in promoting apoptosis, the depletion of this protein via *ABCA12* supports cancer stemness and further contributes to drug resistance [39]. These findings underscore the complex interrelationship between SLs and ABC transporters in modulating breast cancer chemoresistance.

#### 2.2.2 Fatty Acid Metabolism

Fatty acid synthase (FAS), an enzyme involved in *de novo* lipogenesis, is aberrantly upregulated in numerous cancers and is implicated in tumor growth and therapeutic resistance [40]. Notably, the inhibition of FAS suppressed the proliferation of tamoxifen-resistant MCF-7/TamR cells as well as tumor growth in xenograft models [41]. One of the mechanisms underlying FAS-induced drug resistance involves the suppression of intracellular ceramide levels. Meanwhile, doxorubicin has been shown to increase ceramide production via the activation of neutral SMase in MCF-7 breast cancer cells [40]. However, FAS overexpression attenuated this doxorubicin-induced ceramide accumulation, thereby reducing cancer cell apoptosis [40]. This effect is associated with alterations in NF-κB signaling and apoptotic pathways, suggesting that FAS-mediated modulation of SL metabolism may contribute to chemoresistance [40]. Similarly, the induction of apoptosis through FAS inhibition in breast cancer cells was abolished following treatment with the CerS inhibitor fumonisin B1. This finding indicates that ceramide accumulation resulting from FAS inhibition also occurs through the *de novo* ceramide synthesis pathway [42]. Therefore, the role of FAS in mediating chemoresistance and regulating cell death appears to be closely linked to its impact on ceramide biosynthesis.

#### 2.2.3 NF-κB Signaling

NF-κB is a well-established mediator of drug resistance in cancer and can be coordinated with the generation of S1P by SphK1 or SphK2. Moreover, SphK2 inhibition using the selective inhibitor ABC294640 has been shown to suppress cell proliferation and induce apoptosis, at least in part through the downregulation of NF-κB signaling [43]. ABC294640 efficiently reduced cell survival at low micromolar IC_50_ concentrations in both endocrine therapy-resistant MDA-MB-231 cells and chemoresistant MCF-7TN-R breast cancer cell lines [43]. This suggests that pharmacological inhibition of SphK2 may represent a possible strategy to overcome therapeutic resistance in breast cancer. In addition, SphK1 overexpression in TNBC has been implicated in promoting metastasis through NF-κB-mediated transcriptional upregulation of *FSCN1*, a gene known to facilitate metastatic progression [44], consistently indicating a role of S1P in NF-κB activation. While ceramide is generally regarded as a proapoptotic lipid, certain ceramide species—such as C2-ceramide—and the stable overexpression of CerS4 have paradoxically been shown to promote cell survival and proliferation through NF-κB activation [38,45]. Specifically, C2-ceramide may enhance NF-κB signaling via calpain activation, thereby contributing to increased cell viability [45]. Finally, SGMS2 overexpression has been shown to improve breast cancer stemness through NF-κB signaling pathway activation [46,47]. Collectively, these findings suggest that SphK1, SphK2, SGMS2, and CerS4 are functionally linked to NF-κB signaling, with context-dependent effects on cancer cell fate and drug resistance.

### 2.3 The Role of De Novo Ceramide Synthesis in Breast Cancer

#### 2.3.1 CerS in Breast Cancer

Total ceramide levels are elevated in breast cancer, accompanied by increased mRNA expression of *CerS2*, *CerS4*, and *CerS6*, likely contributing to higher C16-, C24-, and C24:1-ceramide levels [48,49]. Notably, elevated C16-ceramide correlates with positive lymph node status, suggesting a role in metastasis [49], while increased C18- and C20-ceramides are observed in ER+ vs. ER− tumors, indicating subtype-specific effects [49]. Distinct ceramide species exert divergent biological functions: long-chain ceramides (C16–C20) generated by CerS4 and CerS6 inhibit proliferation and induce apoptosis in MCF-7 cells, whereas very long-chain ceramides synthesized by CerS2 promote proliferation [50]. Furthermore, chemotherapeutic agents, such as paclitaxel (Taxol) [14], doxorubicin [13], and methotrexate [51], further elevate ceramide levels, particularly C16-ceramide, via *de novo* synthesis to induce apoptosis. These findings highlight the therapeutic potential of targeting *de novo* C16-ceramide synthesis to improve chemotherapy efficacy in breast cancer.

- The functional role of CerS must account for splicing variants. Notably, exon 8 skipping in *CerS2*, observed in luminal B subtype and associated with poor prognosis, produces a catalytically deficient isoform by removing part of the Lag1p motif, reducing very long-chain ceramides (C22–C24) [52]. This reduction was shown to enhance cell proliferation and migration specifically in luminal B subtype [52], which contrasts with reports linking elevated very long-chain ceramides to increased proliferation [50]. These discrepancies underscore the complexity of the CerS2 function and suggest that both expression levels and alternative splicing variants must be considered when evaluating the functional role of CerS in breast cancer.
- CerS2 regulates cell proliferation, invasion and chemoresistance. The expression of CerS2 is higher in low-invasive MCF-7 breast cancer cells than in highly invasive MDA-MB-231 cells. Meanwhile, migration and invasion are suppressed by CerS2 overexpression, whereas CerS2 knockdown enhances these processes [53]. In addition, CerS2 promotes chemosensitivity by inhibiting vacuolar H^+^-ATPase (V-ATPase) activity, leading to alkalinization of the extracellular matrix (ECM) and lysosomes [54,55]. This pH shift attenuates the activity of matrix metalloproteinases (MMP-2 and MMP-9) and ECM degradation, thus impairing tumor invasion [54]. Collectively, CerS2 exerts context-dependent effects in breast cancer, thereby exhibiting therapeutic potential in mitigating invasion and drug resistance.
- The biological effects of CerS depend not only on ceramide acyl chain length but also on the duration and stability of CerS expression. In line with the proapoptotic role of ceramides, transient overexpression of CerS4 or CerS6, which produce long-chain ceramides (C16–C20), suppresses breast cancer cell proliferation and motility [26,50,56]. Conversely, stable CerS4 overexpression in MCF-7 cells activates oncogenic signaling pathways, including Akt/mTOR, β-catenin, and NF-κB, and promotes epithelial-to-mesenchymal transition (EMT) and ABC transporter upregulation [38].

#### 2.3.2 DEGS1 in Breast Cancer

DEGS1 catalyzes the introduction of a double bond at the C4–C5 position in dihydroceramide, converting it into ceramide. Recent studies have identified DEGS1 as a pivotal regulator of anchorage-independent survival, enabling cancer cells to resist apoptosis upon detachment from the ECM [57]. Functioning downstream of HER2-mediated glucose uptake and metabolic reprogramming, DEGS1 supports this survival mechanism and enhances HER2-mediated tumorigenicity [57]. Importantly, elevated DEGS1 expression has been detected in approximately one-third of HER2-positive breast cancer cases and is significantly associated with poor clinical outcomes [57]. These findings indicate DEGS1 as a key effector in HER2-driven metabolic reprogramming and oncogenicity in breast cancer.

### 2.4 Salvage Pathway

#### 2.4.1 Ceramide Glycosylation in Breast Cancer

Glucosylceramide can be further metabolized into complex glycosphingolipids, such as gangliosides and globosides (Fig. 2B).

- The expression of GCS in breast cancer is closely linked to chemoresistance through the regulation of MDR1 expression [58]. Notably, MDR1 overexpression reciprocally enhances GCS expression, establishing a positive feedback loop that reinforces multidrug resistance [59]. GCS overexpression also remodels glycosphingolipid-enriched microdomains, increasing globotriaosylceramide (Gb3) and glucosylceramide levels, which activate Akt and ERK1/2 signaling to upregulate ABCB1 expression [60]. Furthermore, Gb3 elevates cancer stem cell markers, including FGF-2, CD44, and Oct-4, promoting tumorigenesis via the c-Src/β-catenin pathway [61].
- Gangliosides GD2 and GD3 are critical mediators of breast cancer progression (Fig. 2B). GD2 functions as a marker of breast cancer stem cells, while GD3 synthase, the key enzyme in GD2 biosynthesis, drives invasion, EMT, and stemness maintenance [62,63]. GD3 also enhances malignancy by directly activating EGF receptor signaling [64]. Notably, pharmacological inhibition of GD3 synthase with triptolide markedly reduced tumor growth in NOD/SCID mice bearing MDA-MB-231 xenografts, underscoring the therapeutic potential of targeting GD3 [65]. Clinically, elevated GD2 or GD3 synthase expression correlates with poor prognosis across breast cancer subtypes, including TNBC [63,66]. Increased GD2 expression is associated with higher histologic grades, lymph node metastasis, and elevated CD44 and CD24 levels [63,67]. Moreover, anti-GD2 antibody (dinutuximab) treatment effectively eliminated GD2^+^ cells and suppressed TNBC xenograft growth by inhibiting cell adhesion, migration, and mTOR signaling [68]. Collectively, GD2 and GD3 act as oncogenic drivers in breast cancer and represent promising therapeutic targets, particularly in TNBC.
- In addition to GD2 and GD3, other gangliosides contribute to breast cancer pathogenesis, including GM1 and GM3. Overexpression of B3GALT4, the glycosyltransferase responsible for GM1 synthesis, promotes EMT in MCF-10A cells and alters the oncogenic protein profile of small extracellular vesicles [69]. Moreover, Serum ganglioside profiling identified GM3 as a potential biomarker distinguishing luminal B from other breast cancer subtypes [70]. In TNBC models, silencing GM3 synthase in 4T1 cells suppressed migration, invasion, anchorage-independent growth, and lung metastasis [71]. Collectively, these findings highlight the role of glucosylceramide-derived gangliosides in regulating stemness, invasion, and EMT, suggesting that targeting glycosylated ceramide species may represent a therapeutic strategy for aggressive breast cancers such as TNBC.

#### 2.4.2 Sphingomyelin Metabolism in Breast Cancer Signaling and Therapy

Sphingomyelin synthesis is catalyzed by SGMS isoforms: SGMS1, SGMS2, and SGMS-related protein (SGMSr). SGMS1 localizes primarily to the Golgi, while SGMS2 is found in both the Golgi and plasma membrane [72]. SGMSr primarily functions as a ceramide phosphorylethanolamine synthase rather than a canonical sphingomyelin synthase [73]. SGMS1 overexpression inhibited TGF-β1/Smad-induced EMT, as well as the migration and invasion of MDA-MB-231 cells [74]. In contrast, SGMS2 promotes aggressive breast cancer phenotypes by activating TGF-β/Smad signaling, enhancing cancer stemness through NF-κB-mediated upregulation of CD44, ALDH, OCT4, and SOX2 [46,47]. SGMS2 facilitates M2 macrophage polarization, which correlates with poor TNBC prognosis [75]. These findings suggest that SGMS1 and SGMS2 exert opposing roles in breast cancer progression.

Neutral SMase 2 (NSMase2), encoded by *SMPD3*, is mainly located in both the plasma membrane and the Golgi [76], and converts sphingomyelin to ceramide. NSMase2 activation increases C16-, C18-, and C24-ceramide levels, and is upregulated by chemotherapeutics such as daunorubicin and doxorubicin, promoting apoptosis [77,78]. Additionally, NSMase2 regulates exosomal secretion of microRNAs, including miR-210 and miR-10b, which modulate cancer cell invasion, metastasis, and angiogenesis [79,80]. The precise role of NSMase2 in breast cancer tumorigenesis still requires further investigation.

### 2.5 Catabolic Pathway

#### 2.5.1 The Role of Acid CDase in Breast Cancer

Acid CDase (ACDase), encoded by *ASAH1*, catalyzes lysosomal ceramide degradation to sphingosine and fatty acids (Fig. 1). Although high *ASAH1* expression has been associated with a favorable prognosis in several breast cancer contexts, including the ER+ subgroup [81], ductal carcinoma *in situ*, and invasive breast cancer [82], the activation of ACDase in tumors is generally linked to decreased ceramide levels, thereby promoting cancer cell survival. In this context, inhibiting ACDase may exert anticancer effects by elevating intracellular ceramide, thereby enhancing the susceptibility of cancer cells to apoptosis.

Ceranib-2, an ACDase inhibitor, has displayed anticancer effects in MDA-MB-231 and MCF-7 cells by activating apoptotic signaling pathways such as c-Jun N-terminal kinase mitogen-activated protein kinase (JNK) and p38 mitogen-activated protein kinase (MAPK), inhibiting the Akt pathway, and downregulating ERα expression [83]. In another study, ceranib-2 similarly induced cell death through mechanisms including DNA fragmentation, cell cycle arrest, upregulation of proapoptotic proteins Bad and BAX, and downregulation of the antiapoptotic protein Bcl-2 [84]. Another ACDase inhibitor, D-erythro-MAPP, likewise decreased cell viability and induced cell cycle arrest accompanied by alterations in mitochondrial membrane potential [85]. Interestingly, tamoxifen, a selective ERα antagonist, was found to reduce both the ACDase activity and protein levels through a lysosomal cathepsin B-dependent mechanism [86]. Toremifene, an antiestrogen that is structurally similar to tamoxifen, has also been reported as a potent inhibitor of ACDase activity [86]. These findings suggest that tamoxifen may confer therapeutic benefits beyond ERα antagonism, exerting off-target effects through ACDase inhibition.

#### 2.5.2 S1P Metabolism by SphK and Its Effects via S1PR in Breast Cancer

Consistent with the SL rheostat model, in which S1P promotes cell proliferation and survival [2], cytosolic SphK1 and nuclear SphK2—the key enzymes responsible for S1P production—have been implicated in breast cancer progression [87].

• SphK1

Clinical data demonstrated that SphK1 is frequently overexpressed in breast cancer tissues and that SphK1 expression positively correlates with lymph node involvement and distant metastasis [88]. Mechanistically, SphK1 is essential for EGF-induced cell proliferation and migration [89] and promotes cancer cell survival by inhibiting signal transducer and activator of transcription 1 activity [90]. SphK1 expression was shown to be elevated in TNBC; meanwhile, SphK1 inhibition restored cell sensitivity to chemotherapeutic agents such as 5-fluorouracil and doxorubicin [91]. Additionally, SphK1 is associated with the EGF-mediated activation of downstream effectors, including Akt, ERK, and p70S6K [87], and enhanced lung metastasis through the activation of the NF-κB/FSCN1 signaling axis [44]. In addition to modulating cancer-associated signaling pathways, SphK1 has been shown to modulate the membrane localization of HER2; the inhibition of SphK1 promotes the redistribution of HER2 into cytoplasmic punctate structures [92], indicating a role for SphK1 in HER2 trafficking. Finally, the production of S1P by SphK1 has been shown to stimulate both hemangiogenesis and lymphangiogenesis in a murine model of breast cancer metastasis [93].

SphK1 has also been implicated in ER signaling and the development of tamoxifen resistance in breast cancer. Both estradiol and EGF are known to activate SphK1, resulting in increased intracellular S1P production. However, only estradiol can induce the extracellular secretion of S1P via ATP transporters ABCC1 and ABCG2 [94]. Estradiol exerts both rapid and sustained effects on SphK1 activation, which, in turn, modulates intracellular calcium mobilization and ERK1/2 signaling pathways [95]. Additionally, estradiol activates ERα36, a novel ER splice variant, which further enhances SphK1 activity [96]. Although tamoxifen functions as an ERα antagonist, tamoxifen paradoxically acts as an agonist of ERα36, thereby promoting SphK1 expression [96]. Notably, SphK1 is frequently overexpressed in tamoxifen-resistant breast cancer cells, while the pharmacological inhibition of SphK1 has been shown to restore the antiproliferative and proapoptotic effects of tamoxifen. These findings suggest that SphK1 may be applied as a therapeutic target to overcome tamoxifen resistance in breast cancer.

• SphK2

SphK2 was previously considered to play a minimal role in breast cancer; however, emerging evidence has begun to elucidate the functional significance of SphK2, with substantial evidence establishing a pivotal role for SphK1 in breast cancer tumorigenesis. Genetic deletion or pharmacological inhibition of SphK2 has been shown to suppress breast cancer growth and metastasis, in part by attenuating cancer-associated fibroblast (CAF) activation and inhibiting the NF-κB signaling pathway [43,97]. Additionally, treatment with the selective SphK2 inhibitor ABC294640 significantly reduced metastatic potential in TNBC by downregulating key components in the cytoskeletal remodeling pathway, including p21-activated kinase 1 (PAK1), LIM domain kinase 1 (LIMK1), and cofilin1 [98]. These findings suggest that, although the influence of SphK2 may be less pronounced than that of SphK1, SphK2 contributes to breast cancer progression by modulating both intracellular signaling and the tumor microenvironment (TME). Nonetheless, further research is required to delineate the mechanistic roles of SphK2 in breast cancer.

• S1PRs

S1P can be exported from cells and exerts its extracellular functions through binding to a family of five G protein-coupled receptors, S1PR1–5. Among these, S1PR1 has been extensively characterized for its pivotal role in breast cancer progression, contributing to angiogenesis, cell proliferation, survival, and metastasis. The antitumor potential of FTY720 (fingolimod), a functional antagonist of S1PR1, has been demonstrated in breast cancer models. FTY720 initially acts as an S1PR1 agonist, transiently activating downstream signaling; however, upon sustained exposure, FTY720 induces S1PR1 internalization and proteasomal degradation, thereby functioning as a long-term antagonist (Fig. 1) [99]. Indeed, FTY720 administration in a murine model of breast cancer reduced inflammation, S1P signaling, and pulmonary metastasis in E0771 tumor-bearing mice [100]. These effects collectively prolonged overall survival [100], implicating the therapeutic potential of FTY720 in modulating the TME and metastatic progression. Interestingly, enhanced S1PR1 phosphorylation at threonine 236, rather than increased total S1PR1 expression, has been observed in TNBC. Additionally, treatment with either a pan-AKT inhibitor (MK2206) or FTY720 effectively suppressed TNBC cell migration *in vitro* and tumor invasion *in vivo* [101]. Consistent with these findings, the macrophage-specific deletion of S1PR1 in murine models (*S1pr1*^*fl/fl*^ *F4/80*^*Cre/+*^) also prevented pulmonary metastasis in breast cancer and lymphangiogenesis by suppressing interleukin (IL)-1β production and inflammasome activation [102]. Additionally, a monoclonal antibody against S1PR1 (S1PR1-antibody) has demonstrated cytostatic effects in both HER2-positive SK-BR-3 and triple-negative MDA-MB-231 breast cancer cell lines [103], suggesting that direct S1PR1 inhibition has therapeutic potential across molecular subtypes.

In addition to S1PR1, other S1PR family members have also been implicated in breast cancer progression. In a cohort of 304 ER-positive breast cancer patients, immunohistochemical analysis revealed that elevated expression of S1PR1, S1PR3, and ERK1/2 was associated with a shorter time to recurrence and the development of tamoxifen resistance [104], highlighting the potential involvement of these proteins in endocrine therapy failure and disease progression. Furthermore, S1P rapidly increased the expression of SNAI2, a key EMT regulator, via S1PR2 and S1PR3. Meanwhile, stable overexpression of S1PR2 and S1PR3 has been associated with the acquisition of a more aggressive breast cancer phenotype [105]. S1P also promotes cancer stemness via S1PR3-mediated activation of the Notch pathway [106]. Moreover, S1PR3 overexpression has been shown to enhance S1P-induced intracellular calcium mobilization and elevate cyclooxygenase-2 and prostaglandin E2 levels, thereby promoting cancer cell migration [107]. Furthermore, S1PR2 and S1PR3 inhibition or downregulation effectively suppressed tumor cell growth [26,108]. S1P also activates the ERK1/2 signaling pathway through a mechanism that is dependent on both S1PR4 and HER2 signaling [109]. Clinically, high S1PR4 expression has been associated with shorter disease-free survival in patients with ER-negative breast cancer [92]. Collectively, these findings suggest that SphK1-derived S1P exerts oncogenic effects through engagement of not only S1PR1, but also via S1PR2–4; each of which activates distinct downstream signaling that promotes breast cancer progression and therapeutic resistance.

### 2.6 Ceramide-1-Phosphate in Breast Cancer

CERK is reported to be upregulated in metastatic breast cancer cells [110]. Additionally, CERK overexpression has been linked to the activation of oncogenic signaling pathways, including PI3K/Akt/mTOR, RAS/ERK, and RhoA [111]. Functionally, CERK promotes tumor growth, migration, and chemoresistance in TNBC [111] and contributes to the development of tamoxifen resistance [112]. Elevated CERK expression has also been observed in tamoxifen-resistant MCF-7 cells, while the pharmacological inhibition of CERK in these cells led to increased ceramide accumulation, thereby restoring drug sensitivity [113]. Similar to S1P, C1P—the product of CERK activity—plays a crucial role in promoting breast cancer cell survival. Consequently, CERK serves as a key enzyme that counteracts ceramide-induced apoptosis, highlighting its potential as a therapeutic target in breast cancer. Table 1 summarizes the effects of various SL metabolic pathways on breast cancer development and progression.

**Table 1:** The effects of sphingolipid metabolism on breast cancer progression

Genes	SLs changes	Mechanism	Effects on the breast cancer	Ref.
*CerS2*	C24, C24:1-Ceramide ↑	V-ATPase ↓ MMP-2, MMP-9 ↑	Tumor cell invasion ↓	[53]
		V-ATPase ↓	Chemosensitivity ↑ Cell cycle arrest and increased apoptosis after doxorubicin treatment	[54]
		V-ATPase ↓ MMP-2 ↓	Cell growth ↓ Invasion ↓ Metastasis ↓	[55]
*CerS4*	C20-Ceramide ↑	Akt/mTOR, β-catenin, NF-κB, EMT, ABCB1, ABCC1 ↑	Chemoresistance Cell proliferation ↑ Cell migration ↑	[38]
*CerS6*	C16-Ceramide ↑	EMT ↓	Cancer cell motility ↓ Plasma membrane fluidity ↓	[56]
*DEGS1*	Dihydroceramide ↑ Ceramide ↓	Colony formation ↑ Anchorage-independent survival ↑ HER2-driven glucose metabolism ↑	Tumorigenicity ↑ Aggressiveness ↑	[57]
*GCS (UGCG)*	Glucosylceramide ↑	Akt, ERK1/2, ABCB1 ↑	Chemoresistance	[60]
		CD44, c-Src/β-catenin ↑	Stemness ↑	[61]
*B3GALT4*	GM1 ↑	EMT ↑ Vesicular GM1 ↑ Small extracellular vesicles ↑	Migration ↑	[69]
*GM3 synthase*	GM3 ↑	PI3K/Akt ↓	Anchorage-independent growth ↑ Migration ↑ Invasion ↑	[71]
*GD3 synthase*	GD3 ↑	PI3/Akt, ERK, c-Met ↑	Cell proliferation ↑ Tumor growth ↑	[62]
		EMT, migration, invasion	Metastasis ↑	
	GD2 ↑	EMT, CD44 ↑	Stemness ↑ Cell proliferation ↑ Tumor growth ↑	[65]
		Lymph node invasion Larger size of tumors	More aggressive tumor	[63]
		Akt, ERK, c-Met ↑	Tumorigenicity ↑ Aggressiveness ↑	[67]
*SphK1*	S1P ↑	NF-κB and FSCN1 ↑	Metastasis ↑	[44]
		STAT1 ↓	Cell proliferation ↑ Mammosphere formation ↑	[90]
			Hemangiogenesis lymphangiogenesis	[93]
		Notch ↑	Migration ↑ Invasion ↑	[91]
		Akt, ERK, p38 ↑	Cell growth ↑ Cell survival ↑	[87]
*SphK2*	S1P ↑	NF-κB ↑, Ki67 ↑	Drug resistance ↑ Cell proliferation ↑	[43]
		p53↓, cancer-associated fibroblasts activation	Tumor growth ↑ Lung metastasis ↑	[97]
		PAK1, LIMK1, Cofilin1	Metastasis	[98]
	S1P binding	Inflammasome component Nlrp3, IL-1β ↑	Lung metastasis Lymphangiogenesis	[102]
		ERK1/2	Tamoxifen resistance	[104]
*S1PR1*		p-Akt, p-ERK, p-Stat3, p-p65 ↑	Inflammatory cytokine ↑ Macrophage infiltration ↑ Tumor progression ↑ Lung metastasis	[100]
	S1PR1 (T236) phosphorylation ↑		TNBC migration ↑ TNBC invasion ↑	[101]
*S1PR2*	S1P binding	p-Akt, p-S6K ↑	Cell proliferation ↑	[26]
		YAP, SNAI2, EMT ↑	Invasion	[105]
*S1PR3*	S1P binding	ALDH, Notch ↑	Stemness ↑	[106]
		COX2, PGE2 synthase ↑	Migration ↑	[107]
*S1PR4*	S1P binding	p-ERK, tyrosine phosphorylation of HER2		[109]
*ACDase*	Ceramide degradation	Ceramide ↓	Better prognosis	[81,82]
*SGMS1*	Sphingomyelin synthesis	TGF-β/Smad, EMT ↓ E-cadherin ↑, Vimentin ↓	Migration ↓ Invasion ↓	[74]
*SGMS2*	Sphingomyelin synthesis	TGF-β/Smad ↑	Proliferation ↑ Invasion ↑ Apoptosis ↓	[114]
		NF-κB, CD44, ALDH, OCT4, SOX2 ↑	Stemness ↑	[47]
*NSMase2*	Sphingomyelin degradation to ceramide	Secretion of exosomal miRNA (miR-201, miR-10b)	Invasion ↑ Metastasis ↑ Angiogenesis ↑	[79,80]
*CERK*	C1P	PI3K/Akt/mTOR, RAS/ERK, RhoA ↑	TNBC growth ↑ Migration ↑ Chemoresistance	[111]
		PI3K/Akt ↑, p-p53, cleaved caspase-3 ↓	Tamoxifen resistance Cell growth ↑ Invasion ↑	[112]
Short-chain ceramide	C2-ceramide	Akt ↓	Cell proliferation ↓ Apoptosis ↑	[115]
		Ceramide channel formation in mitochondria	Cell death ↑	[27]
	C6-ceramide	Mitochondrial permeability, ROS generation, AMPK, JNK ↑	Cell proliferation ↓ Apoptosis ↑	[116]
	C8-ceramide	MDR1 ↑		[117]

Note: Abbreviation: ABC, ATP binding cassette; ACDase, acid ceramidase; Akt, protein kinase B; ALDH, aldehyde dehydrogenase; CERK, ceramide kinase; CerS, ceramide synthase; COX2, cyclooxygenase-2; C1P, ceramide-1-phosphate; DEGS1, dihydroceramide desaturase 1; EMT, epithelial-mesenchymal transition; ERK, Extracellular signal-Regulated Kinases; FSCN1, fascin actin-bundling protein 1; GCS, glucosylceramide synthase; HER2, human epidermal growth factor receptor 2; IL, interleukin; LIMK1, LIM Domain kinase 1; MDR1, multidrug resistance 1; MMP, matrix metallopeptidase; mTOR, mammalian target of rapamycin; NF-κB, Nuclear Factor kappa-light-chain-enhancer of activated B cells; nlrp3, NLR family, pyrin domain containing 3; Notch, neurogenic locus notch homolog protein; NSMase2, neutral sphingomyelinase 2; PAK1, p21 activated kinase 1; PGE2, prostaglandin E2; PI3K, phosphatidylinositol 3-kinase; RhoA, Ras homolog family member A; SGMS, sphingomyelin synthase; SNAI2, Snail family transcriptional repressor 2; Sphk, sphingosine kinase; STAT1, Signal transducer and activator of transcription 1; S1P, sphingosine-1-phosphate; S1PR, S1P receptor; TGF, transforming growth factor; YAP, yes-associated protein.

## 3 The Effects of Chemotherapy on SL Metabolism in Breast Cancer

### 3.1 Doxorubicin and Daunorubicin

Doxorubicin has been reported to upregulate the transcription of GCS expression in MCF-7 cells [118]. In contrast to these findings, another study demonstrated that doxorubicin treatment can inhibit GCS activity in MCF-7 cells, reducing hexosylceramide levels [119]. Moreover, the study revealed a dynamic shift in SL composition following doxorubicin treatment, characterized by increased sphingosine, S1P, dihydroceramide, and ceramide levels, accompanied by a concomitant decrease in DEGS activity [119]. Additionally, doxorubicin has been shown to upregulate *NSMase2* transcription, and this upregulation, which is mediated in a p53-dependent manner, is essential for doxorubicin-induced cell death [77]. Similar to doxorubicin, daunorubicin enhances ceramide generation via CerS through *de novo* ceramide synthesis [120], and also by upregulating neutral SMase activity, particularly through NSMase2 [78].

### 3.2 Tamoxifen

**Figure 4:**
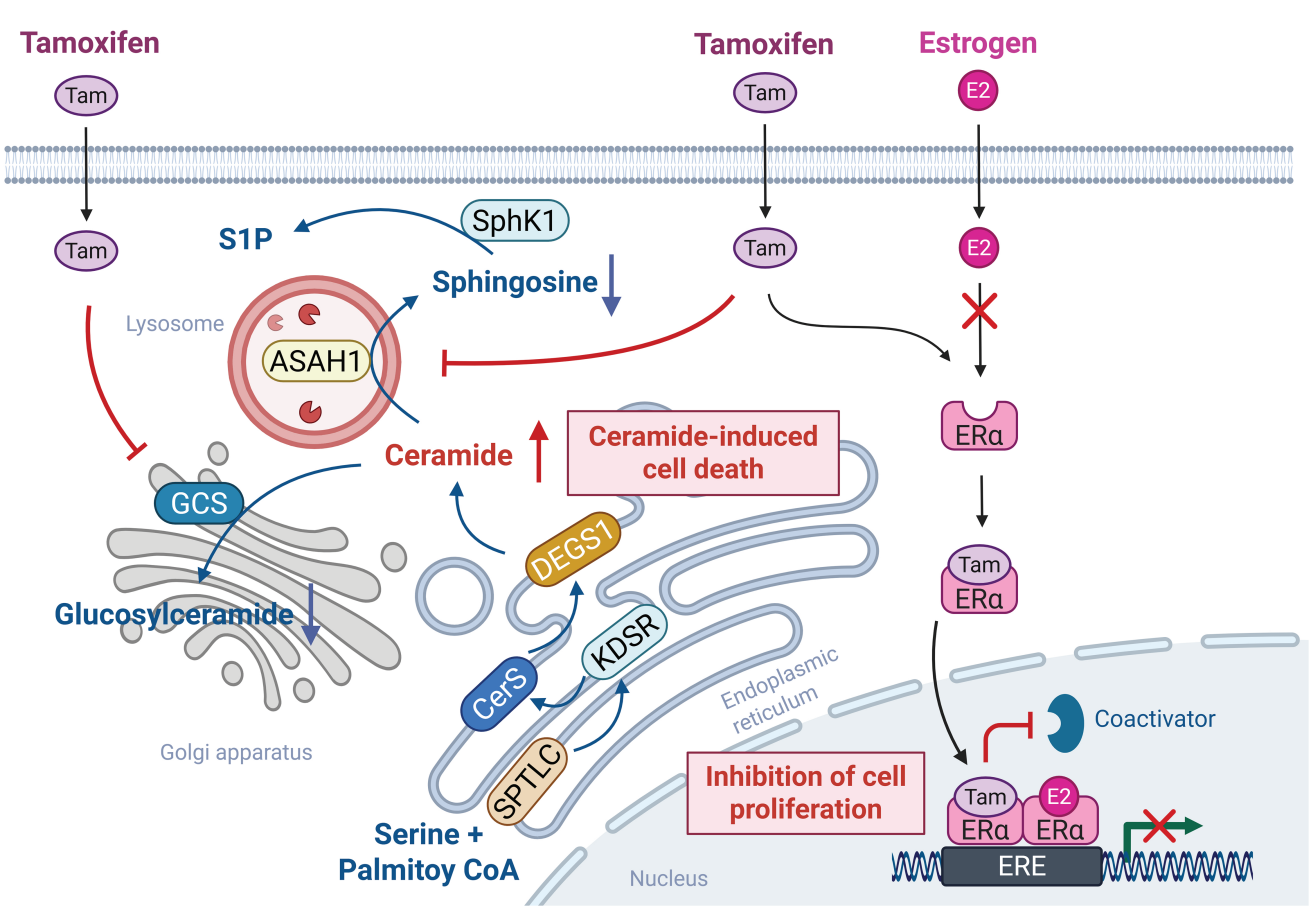
Tamoxifen-mediated off-target SL-modulating effects. Tamoxifen primarily functions as a selective estrogen receptor modulator by competitively binding to estrogen receptor alpha (ERα), thereby inhibiting estradiol (E2)-driven transcription and cell proliferation. Meanwhile, in addition to the role of tamoxifen in estrogen signaling, tamoxifen also exerts off-target effects on SL metabolism. Specifically, tamoxifen inhibits acid ceramidase (ASAH1) and glucosylceramide synthase (GCS), promoting ceramide accumulation. This elevation in ceramide levels enhances ceramide-induced apoptotic signaling, contributing to the overall anticancer efficacy of tamoxifen. The figure was created with BioRender.com, accessed on 03 July 2025. Abbreviation: ERα, estrogen receptor alpha, Tam, tamoxifen

Tamoxifen, a selective ER modulator, functions as an ER antagonist and is widely used as a first-line therapy for ER-positive breast cancer [4]. Meanwhile, tamoxifen has also been shown to modulate SL metabolism in addition to its role in hormonal signaling. Specifically, tamoxifen inhibits the activities of GCS and ACDase, thereby reducing glucosylceramide synthesis and ceramide hydrolysis [4]. Given that both GCS and ACDase are implicated in the development of multidrug resistance in breast cancer, tamoxifen, which promotes intracellular ceramide accumulation, may enhance cancer cell susceptibility to apoptosis as well as overcome therapeutic resistance (Fig. 4). These properties suggest that tamoxifen may synergize with chemotherapeutic agents that induce ceramide generation. Indeed, combination therapies involving tamoxifen and agents, such as β-sitosterol [121] or doxorubicin [122], have demonstrated enhanced anticancer efficacy, likely mediated through increased ceramide levels and amplified apoptotic signaling.

### 3.3 Paclitaxel and Fenretinide

Paclitaxel (Taxol) is an anticancer agent that modulates microtubule dynamics and is commonly used as a first-line treatment for breast cancer [123]. Paclitaxel, which stabilizes microtubule structures, inhibits cell division and proliferation, leading to cell death [123]. Interestingly, paclitaxel also enhances ceramide production by activating SPTLC, thereby inducing apoptosis in human breast cancer cells [14,124].

The cytotoxic effect of fenretinide [N-(4-hydroxyphenyl)retinamide, 4-HPR] has been reported to be mediated through a redox-sensitive mechanism involving elevated ceramide levels in MCF-7 cells [15] and sphinganine accumulation in the doxorubicin-resistant MCF-7/AdrR cells [125].

### 3.4 Fingolimod (FTY720) and Opaganib (ABC294640)

The U.S. Food and Drug Administration has approved the use of FTY720 for the treatment of multiple sclerosis [126]. Additionally, numerous preclinical studies have demonstrated that FTY720 exhibits anticancer efficacy in breast cancer models; however, FTY720 has yet to receive official clinical approval for breast cancer therapy [100,126,127]. FTY720 has been reported to induce apoptosis, suppress tumor growth, and inhibit metastasis in TNBC models, primarily through antagonistic effects on the S1PR [127]. In addition, FTY720 inhibits cellular proliferation by targeting SphK1 and promoting apoptosis by downregulating the PI3K/AKT and MAPK signaling pathways, thereby enhancing the radiosensitivity of breast cancer cells [126]. Furthermore, FTY720 exhibits potential antimetastatic activity by disrupting the SphK1/S1P/S1PR1 axis, a key signaling pathway implicated in breast cancer progression and metastasis [100].

ABC294640 is a selective SphK2 inhibitor that exhibits minimal activity against SphK1 [128]. Pharmacological inhibition with ABC294640 resulted in a significant reduction in intracellular S1P levels and a concomitant accumulation of ceramide species, including C16-ceramide, in both stromal fibroblasts and breast cancer cells [97,128]. These alterations in SL metabolism disrupted cell survival and proliferation, enhanced apoptotic signaling, and suppressed NF-κB-mediated pro-survival pathways, collectively contributing to inhibited tumor growth [43,97,128].

### 3.5 NVP-231

**Figure 5:**
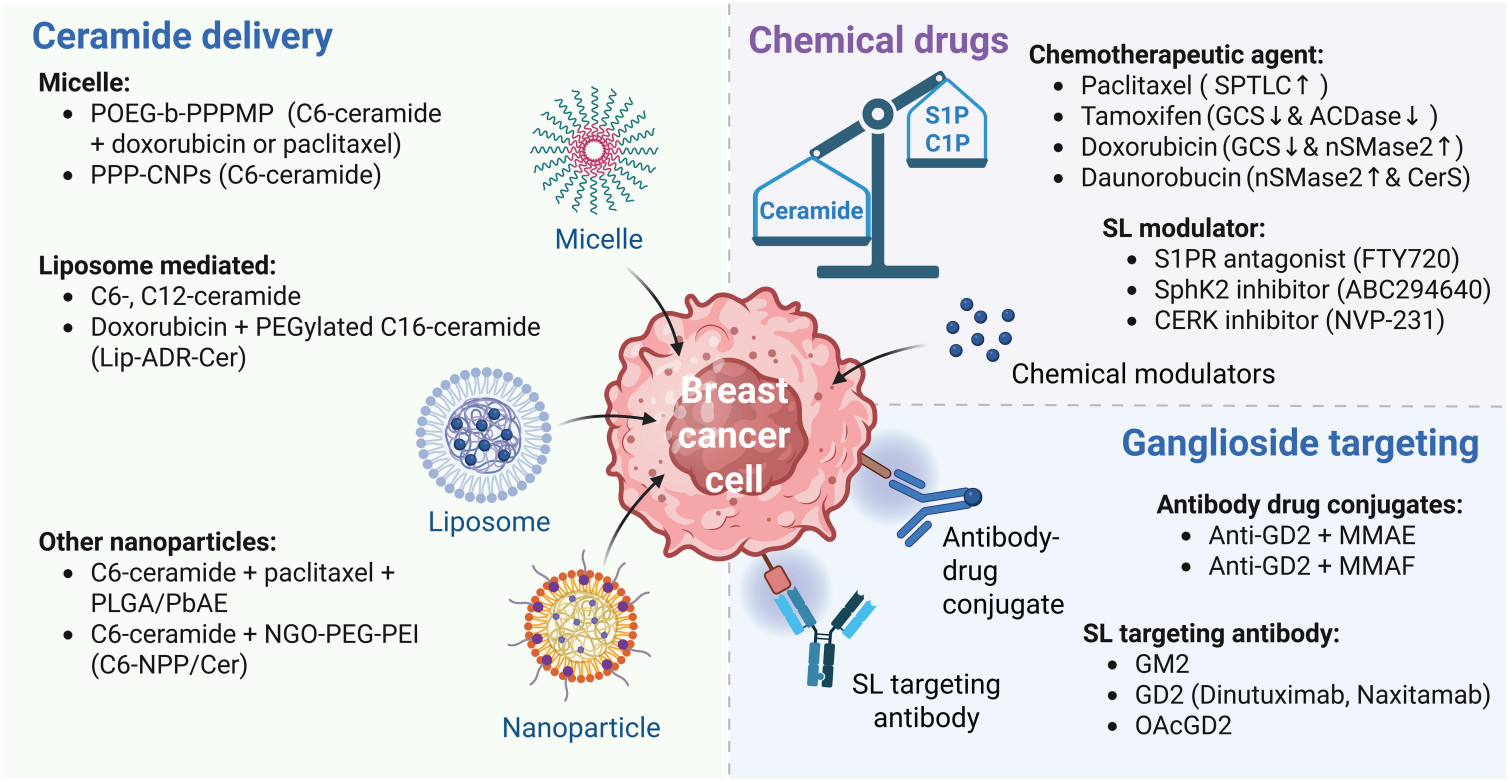
SL-based therapeutic strategies for breast cancer. Therapeutic approaches targeting SL metabolism in breast cancer encompass multiple strategies. Intracellular delivery of ceramide can be facilitated through nanocarrier systems such as micelles, liposomes, and various nanoparticle platforms, enhancing stability, bioavailability, and tumor selectivity. Meanwhile, chemotherapeutic agents, including paclitaxel, tamoxifen, doxorubicin, and daunorubicin, have also been shown to modulate SL metabolism. In addition, small-molecule agents that modulate the transcription or enzymatic activity of key enzymes in the SL metabolic pathway, such as sphingosine kinases (SphK), acid ceramidases (ACDase), and glucosylceramide synthase (GCS), represent a promising avenue for intervention. Furthermore, gangliosides (e.g., GM2 and GD2), which are highly expressed in certain breast cancer subtypes, can be therapeutically targeted using monoclonal antibodies or antibody–drug conjugates, thereby offering potential for selective cytotoxicity against tumor cells. The figure was created with BioRender.com, accessed on 03 July 2025. Abbreviation: MMAE, monomethyl auristatin E; MMAF, monomethyl auristatin F; NGO-PEG-PEI; polyethylene glycol (PEG) and polyethylenimine (PEI) co-conjugated ultra-small nano-GO; OAcGD2, O-acetylated GD2; PLGA/PbAE, poly(_D,L_-lactide-*co*-glycolide)/poly(beta-amino ester); POEG-*b*-PPPMP, PPMP prodrug-based polymeric nanocarrier; PPP-CNP, ceramide-loaded POEG-*b*-PPPMP micelles; SL, sphingolipid

CERK plays a pivotal role in tamoxifen resistance, and its inhibition by NVP-231 raises ceramide levels and concomitantly reduces C1P levels [113,129]. NVP-231 selectively reduces viability and induces apoptotic cell death in tamoxifen-resistant cells (MCF-7-HER2, MCF-7-TAM1, and BT474) to a higher magnitude than in tamoxifen-sensitive cells (MCF-7 and T47D cells) [113]. This confirmed that tamoxifen-resistant breast cancer cells are more sensitive to CERK inhibition [113]. This sensitivity was corroborated in patient-derived xenograft organoid models, where NVP-231 significantly inhibited the growth of the fulvestrant-resistant HCI-011-FR model compared to the fulvestrant-sensitive ER+ HCI-011 model. Mechanistically, C8-ceramide treatment induced significantly higher cell death in tamoxifen-resistant MCF-7-HER2 and MCF-7-TAM1 cells compared to parental MCF-7 cells [113]. This suggests that the tamoxifen-resistant cells are more sensitive to ceramide-induced cell death compared to tamoxifen-sensitive cells [113]. Additionally, NVP-231 induced M-phase cell cycle arrest in MCF-7 cells [129] and suppressed the motility and invasiveness of breast cancer cells by modulating the PI3K/Akt and Rho kinase signaling pathways [110]. The effects of these aforementioned drugs are summarized in Table 2 and Fig. 5.

**Table 2:** Modulation of sphingolipid pathways by chemotherapeutic drugs

Chemotherapy	Mechanism	Sphingolipids change	Ref.
Doxorubicin	*De novo* ceramide synthesis↑ NSMase ↑ Dihydroceramide desaturase activity ↓	Ceramide ↑ Glucosylceramide ↑ Sphingosine ↑ S1P ↑ Dihydroceramide ↑	[118,119]
Daunorubicin	*De novo* ceramide synthesis↑ NSMase ↑	Ceramide ↑	[124]
Tamoxifen	GCS inhibitor ACDase inhibitor	Ceramide ↑	[4]
Paclitaxel (Taxol)	*De novo* ceramide synthesis↑	Ceramide ↑	[14,124]
Fenretinide	ROS ↑	Ceramide ↑	[15,125]
	Cell growth ↓	Dihydroceramide ↑	
	Apoptosis↑	Sphinganine ↑	
Fingolimod (FTY720)	SphK1 ↓	S1P ↓	[100,126]
Opaganib (ABC294640)	SphK2 ↓	S1P ↓	[97,128]
		Ceramide ↑	
		C16-dihydroceramide ↑	
NVP-231	CerK inhibitor	Ceramide ↑	[129]
		C1P ↓	

Note: Abbreviation: ROS, reactive oxygen species.

## 4 Treatment with Short-and Medium-Chain (C2–C8) Ceramides as a Breast Cancer Therapy

Unlike long-chain and very long-chain ceramides (C16–C30), which are the predominant physiological forms in mammals, short- and medium-chain ceramides (C2–C8) possess higher membrane permeability, allowing them to be readily internalized and processed by cells [130]. As such, the anticancer effects of short- and medium-chain ceramides have been relatively well established *in vitro*. Indeed, C2-ceramide has been shown to significantly inhibit proliferation and induce apoptosis in breast cancer cells [115] and induce mitochondrial channel formation, leading to cell death [27]. Meanwhile, the co-treatment of C2-ceramide and doxorubicin elicited more pronounced antiproliferative and proapoptotic effects compared to doxorubicin alone [131]. Similarly, C6-ceramide markedly enhanced docetaxel-induced growth inhibition and apoptosis in MCF-7 and MDA-MB-231 cells [116]. This enhancement was associated with mitochondrial permeability transition pore opening, increased ROS production, and the activation of proapoptotic signaling pathways including AMPK and JNK [116]. Supporting these findings, an *in vivo* study demonstrated that systemic intravenous administration of C6-ceramide in a PEGylated liposomal formulation significantly suppressed solid tumor growth in a syngeneic BALB/c mouse model of breast adenocarcinoma [132], underscoring the potential anti-neoplastic efficacy of C6-ceramide.

However, despite these promising effects, prolonged exposure to short- and medium-chain ceramides may induce resistance mechanisms. For instance, C2-ceramide treatment has been reported to activate NF-κB-dependent survival signaling via calpain activation [45]. Similarly, C6-ceramide treatment increased the expression of MDR1 and GCS, both of which contribute to chemoresistance [133]. Likewise, C8-ceramide treatment also upregulated MDR1 expression [117]. Additionally, the phosphorylated derivatives of short-chain ceramides, such as C2-ceramide-1-phosphate and C8-ceramide-1-phosphate, have been shown to enhance DNA synthesis and promote cell proliferation [134], suggesting that CERK-mediated metabolism of short- and medium-chain ceramides could negate their cytotoxic effects. Given the complex and dynamic metabolism of ceramides—which can generate various bioactive lipids with opposing effects on cancer cell survival—therapeutic strategies employing short- and medium-chain ceramides require careful and comprehensive investigation before clinical application. Indeed, despite the diverse biological impacts reported, a phase II study of topical ceramides for cutaneous breast cancer showed minimal or negligible therapeutic outcomes [135].

## 5 Sphingolipid-Based Therapy

### 5.1 Ceramide Delivery System in Breast Cancer: A New Strategy

The co-administration of ceramide with chemotherapeutic agents through various delivery platforms, including liposomes, micelles, nanoparticles, and other nanocarrier systems, has demonstrated enhanced antitumor efficacy (Table 3; Fig. 5). Liposomes, micelles, and nanoparticles are all nanoscale drug delivery systems, yet these systems differ significantly in composition, structure, and drug-loading mechanisms [136]. Liposomes are phospholipid-based vesicular structures composed of one or more lipid bilayers surrounding an aqueous core, allowing encapsulation of both hydrophilic and hydrophobic drugs [137]. In contrast, micelles are spherical assemblies formed by amphiphilic surfactant molecules, with hydrophobic cores capable of solubilizing lipophilic compounds [138]. Nanoparticles, a broader category, encompass a wide range of materials, including polymers, lipids, and inorganic substances, with variable architecture and surface properties, thereby enabling diverse strategies for drug delivery and targeting [139].

**Table 3:** Ceramide delivery system

Nanocarriers	Delivery system	Ceramide	Chemotherapy	Effect	Mechanism	Ref.
Liposome	PEGylated liposome	C6-ceramide	N/A	Suppressed the growth of solid tumors	Inhibited microvessel network formation and cellular proliferation	[132]
	Nanoliposome (NaL-C6)	C6-ceramide	N/A	Reduced proliferation of cancer cells	Inhibited endothelial cell adhesion	[140]
	Nanoliposome	C6-ceramide	Tamoxifen	Amplified the antiproliferative effect of C6-ceramide	Increased C6-ceramide-induced apoptosis by inhibiting acid ceramidase	[141]
	PEGylated liposome	C6-ceramide C12-ceramide	Doxorubicin (DOX)	Marked reduction in the viability of cancer cells. Enhanced cytotoxicity of doxorubicin	Enhanced liposomal doxorubicin toxicity in a cell line-dependent manner	[142]
	Lip-ADR-C16 (PEGylated liposome)	C16-ceramide	Adriamycin (doxorubicin)	Enhanced cytotoxicity and antitumor activity	N/A	[144]
	F3-peptide-targeted liposome	C6-ceramide	Doxorubicin	Enhanced cytotoxicity even at lower doses of doxorubicin	Cytotoxicity varied depending on cell-level delivery route	[143]
Micelle	POEG-*b*-PPPMP	C6-ceramide	Doxorubicin and paclitaxel	Enhanced the anticancer activity of the drugs	Suppressed *GCS* mRNA expression. Accumulation of proapoptotic ceramide	[146]
	PPP-CNP	C6-ceramide	DM102(ACDase inhibitor) and trastuzumab	Suppressed cell proliferation. Promoted apoptosis	Increased intracellular ceramide concentration	[147]
Nanoparticle	Linear-dendritic nanoparticle	C6-ceramide	N/A	Inducted apoptosis. Inhibited cell proliferation	Temperature-dependent ceramide accumulation	[148]
	PLGA/PbAE	C6-ceramide	Paclitaxel	Induced cell apoptosis	Prolonged plasma retention of PTX. Accumulation of ceramide at the tumor site	[149]
	C6-NPP/Cer (C6-ceramide-loaded NGO-PEG-PEI)	C6-ceramide	N/A	Enhanced antitumor effects	Improved cellular uptake and apoptosis. Inhibited cell proliferation	[150]

Note: Abbreviation: N/A: not applicable; NGO-PEG-PEI; polyethylene glycol (PEG) and polyethylenimine (PEI) co-conjugated ultra-small nano-GO; PLGA/PbAE, poly(_D,L_-lactide-*co*-glycolide)/poly(beta-amino ester); POEG-*b*-PPPMP, PPMP prodrug-based polymeric nanocarrier; PPP-CNP, ceramide-loaded POEG-*b*-PPPMP micelles; SL, sphingolipid.

#### 5.1.1 Liposome-Based Delivery Systems

Liposomal ceramide formulations have promoted enhanced stability, tumor-targeting capacity, and cytotoxic efficacy compared to ceramide alone. Liposomal C6-ceramide delivery significantly enhanced dose-dependent cytotoxicity in murine 410.4 mammary adenocarcinoma cells compared to the non-liposomal (free) form of C6-ceramide [132]. Moreover, intravenous administration of PEGylated liposomal C6-ceramide inhibited microvessel formation and suppressed cell proliferation, thereby exhibiting potent antitumor activity in a human xenograft model established using MDA-MB-231 breast adenocarcinoma cells [132]. Similarly, C6-ceramide nanoliposomes suppressed tumor metastasis by activating tumor-suppressive signaling pathways, including PI3K and PKCζ, and by modulating integrin affinity in MDA-MB-231 breast cancer cells [140]. Furthermore, synergistic therapeutic efficacy was observed following the co-treatment of cell-permeable nanoliposomal ceramide and an anticancer agent. For example, tamoxifen enhanced the antiproliferative effects of nanoliposomal C6-ceramide through combined targeting of cell cycle progression, as well as lysosomal and mitochondrial integrity [141]. Furthermore, the proapoptotic effect of C6-ceramide was amplified by tamoxifen-induced lysosomal destabilization and potentially through ACDase inhibition, reducing S1P [141]. Similarly, when the cytotoxic effects of doxorubicin encapsulated in liposomes containing C6- or C12-ceramide were evaluated in HeLa, HCT116, and MDA-MB-231 cells, the liposomal doxorubicin toxicity was enhanced by the ceramide species in a cell line-specific manner. Notably, C6-ceramide promoted the greatest effect in HeLa and HCT116 cells, while C12-ceramide exhibited the strongest enhancement in MDA-MB-231 cells [142]. F3-targeted liposomes encapsulating doxorubicin: C6-ceramide (1:2 molar ratio) significantly increased apoptosis in drug-resistant/triple-negative MDA-MB-231 breast cancer cells [143]. Moreover, the antitumor efficacy of novel nanoliposomes that co-delivered doxorubicin and PEGylated C16-ceramide (Lip-ADR-Cer) was superior in mice bearing MCF-7/ADR xenograft tumors to that of conventional nanoliposomes lacking PEGylated C16-ceramide (Lip-ADR) [144]. A clinical study on ceramide nanoliposomes demonstrated considerable stability and confirmed the associated pharmacokinetics [145], opening the possibility for future use with other drugs. However, confirming conclusions is premature, and further clinical studies are required.

#### 5.1.2 Micelle-Based Delivery Systems

Micelle-based ceramide delivery systems have also exhibited enhanced cytotoxicity and the ability to overcome drug resistance. Recent studies have shown that micelle-based delivery using POEG-*b*-PPPMP—a polymer synthesized via one-step reversible addition-fragmentation chain transfer (RAFT) polymerization from a PPMP monomer—improves anticancer efficacy by co-delivering C6-ceramide and chemotherapeutic agents such as doxorubicin and paclitaxel [146]. These micelles consist of a hydrophilic POEG block and a hydrophobic PPMP block [146]. Functioning as a prodrug system, POEG-*b*-PPPMP micelles release PPMP, which suppresses *GCS* mRNA expression [146]. The study demonstrated that co-delivery of doxorubicin with POEG-*b*-PPPMP not only inhibited the doxorubicin-induced upregulation of *GCS* but also increased the accumulation of proapoptotic ceramides and enhanced cytotoxicity in tumor cells, outperforming other delivery systems, including POEG-*b*-POM and doxil (liposomal doxorubicin) [146]. In another study, PPP-CNPs—ceramide-loaded nanoparticles categorized as micelles—facilitated the selective uptake of C6-ceramide by cancer cells, resulting in elevated intracellular ceramide levels and the subsequent induction of cancer cell death [147].

#### 5.1.3 Nanoparticles-Based Delivery Systems

Finally, nanoparticles have been applied to ensure delicate adjustments in the delivery of ceramide. For example, the delivery of C6-ceramide using linear-dendritic, thermosensitive, and biodegradable nanoparticles resulted in temperature-dependent intracellular accumulation, leading to enhanced apoptosis and inhibition of cell proliferation [148]. In another approach, co-delivery of C6-ceramide and paclitaxel via PLGA/PbAE (poly(_D,L_-lactide-*co*-glycolide))/poly(beta-amino ester)-blend nanoparticles effectively prolonged the plasma retention of paclitaxel and increased tumor accumulation of C6-ceramide, thereby significantly improving therapeutic efficacy [149]. In this dual-delivery system, paclitaxel was encapsulated within the pH-responsive, rapidly releasing polymer PbAE, while C6-ceramide was incorporated into the slow-releasing polymer PLGA [149]. Interestingly, this design allowed for the controlled release of the drug, wherein paclitaxel was rapidly released under the acidic conditions of the TME, while C6-ceramide exhibited sustained release, promoting apoptosis [149]. Subsequently, the formulation enhanced the intratumoral residence time of both agents while reducing systemic clearance [149]. Another promising strategy involved the use of ceramide–graphene oxide nanoparticles, such as C6-NPP/Cer (C6-ceramide-loaded NGO-PEG-PEI), which effectively enhanced the ceramide anticancer activity [150]. Treatment with C6-NPP/Cer significantly improved cellular uptake, inhibited proliferation, and induced apoptosis in breast cancer cells [150]. Meanwhile, C6-NPP/Cer reduced tumor growth and increased apoptotic cell death in both the *in vitro* and *in vivo* experiments [150].

In conclusion, incorporating ceramide with conventional anticancer agents through advanced delivery platforms, such as liposomes, micelles, and nanoparticles, has demonstrated synergistic therapeutic effects. These strategies have exhibited not only enhanced treatment efficacy but also offer the potential to reduce the required doses of conventional chemotherapeutics, thereby minimizing associated toxicities. Furthermore, such delivery systems can improve the targeted delivery and bioavailability of therapeutic agents, representing a promising approach for more efficient and effective breast cancer treatments.

### 5.2 Antibody Therapy Against SLs in Breast Cancer

GM2 and GD2 are major gangliosides overexpressed on the surface of various solid tumors, including breast cancer, and have emerged as promising targets for immunotherapeutic strategies. Several monoclonal antibodies targeting GM2 and GD2 have demonstrated potent antitumor activity (Fig. 5). For instance, in the GM2-expressing SBC-3 human small cell lung cancer cell line, KM966—a mouse/human IgG1 chimeric anti-GM2 antibody—exerted strong antitumor activity through Fc receptor-mediated antibody-dependent cell-mediated cytotoxicity (ADCC) [151]. Notably, KM966 also induced cytotoxicity in adriamycin-resistant human breast cancer cells (MCF-7/AdrR), suggesting that passive immunotherapy with KM966 may offer a promising strategy to overcome adriamycin resistance in breast cancer [152].

GD2, which is abundantly expressed on breast cancer stem-like cells—a subpopulation implicated in tumor progression and chemoresistance—has emerged as a critical therapeutic target. The anti-GD2 monoclonal antibodies such as dinutuximab (Unituxin) and naxitamab (Danyelza, hu3F8), approved by the U.S. Food and Drug Administration in 2015 and 2020, respectively, have been employed to enhance cytotoxic responses against GD2^+^ breast cancer stem-like cells [58,144]. These anti-GD2 antibodies significantly increase ADCC, particularly when combined with natural killer cells activated by NKTR-255, a polymer-conjugated IL-15 receptor agonist. This combination therapy leads to improved cytotoxicity and enhanced therapeutic durability [153]. However, a notable clinical limitation remains the expression of GD2 on healthy peripheral nerve fibers, which has been associated with adverse effects such as allodynia. Thus, this poses challenges for the widespread clinical application of anti-GD2 antibody therapy [154,155].

Another emerging strategy involves the use of GD2-targeted antibody–drug conjugates (ADCs). Indeed, ADCs constructed by conjugating anti-GD2 antibodies with monomethyl auristatin E (MMAE) or monomethyl auristatin F (MMAF), potent microtubule-disrupting agents, retained their antigen-binding specificity and showed cytotoxic effects proportional to GD2 expression levels (Fig. 5). Moreover, MMAF-conjugated ADCs were highly effective in GD2-overexpressing cells, while MMAE-based ADCs also exhibited moderate cytotoxicity in cells with lower GD2 expression [156].

Interestingly, modulating ceramide metabolism has also been explored as a method of further potentiating GD2-targeted therapies. Notably, O-acetylated GD2 (OAcGD2), a tumor-selective variant of GD2, is negatively regulated by CERK (Fig. 5) [157]. Thus, the pharmacological inhibition of CERK increases OAcGD2 expression and attenuates cell migration; however, this intervention alone cannot elicit significant apoptosis in MDA-MB-231 cells overexpressing GD3 synthase (MDA-MB-231 GD3S^+^) [157]. Comparatively, a marked enhancement in ADCC was observed when CERK inhibition was combined with natural killer cells and anti-OAcGD2 monoclonal antibodies [157]. These findings suggest that targeting CERK in conjunction with immunotherapy could represent a promising combinatorial strategy for improving the therapeutic outcomes of GD2-based interventions in breast cancer.

### 5.3 SL-Based Immune Therapy-Managing Tumor Microenvironment and CAR-T Cell Therapy

The TME comprises tumor cells and various non-neoplastic stromal components, including immune cells. These cells interact through direct contact or via soluble mediators, collectively promoting angiogenesis, invasion, and metastasis. Among SLs, S1P exerts predominant effects on the TME. Apoptotic tumor cell-derived S1P drives macrophage polarization toward an M2-like phenotype by suppressing NF-κB activation [158]. Moreover, S1P activates tyrosine kinase receptor A, triggering PI3K/AKT and p38 MAPK signaling to induce IL-10 and other proinflammatory cytokines, thereby promoting the tumor-supportive phenotype of macrophages [159]. Pharmacological modulation of S1P signaling, using the SphK1 inhibitor SK1-I [93], the SphK2 inhibitor ABC294640 [97], the S1P modulator FTY720 [100], the S1P scavenger NOX-S93 [160], or the anti-S1P monoclonal antibody Sphingomab^®^ [161], provides a potential approach to remodel the TME.

Recently, CAR-T cell therapy has emerged as a promising approach, involving the *ex vivo* expansion of T cells engineered to recognize specific tumor antigens and the reinfusion of these cells into patients. GD2, which is highly expressed on the surface of breast cancer cells, has been targeted in CAR-T therapy. Preclinical studies indicate that GD2-specific CAR-T cells traffic effectively to both primary tumors and metastatic sites, reducing lung metastasis [162]. However, similar to GD2 antibody therapy, this approach carries neurological risks, including potential encephalitis at high doses [163]. Additional tumor-associated gangliosides, such as OAcGD2, GM1, and GM3, and S1PRs have also been proposed as CAR-T targets.

SL signaling may further enhance CAR-T therapy. For instance, the inhibition of S1PR3 improves CAR-T cell antitumor activity by alleviating T cell exhaustion and remodeling the TME via the recruitment of proinflammatory macrophages [164]. Thus, SL modulation can serve both as a direct CAR-T target and as a supportive strategy to augment efficacy.

### 5.4 Future Perspectives and Limitations of SL-Based Therapy

SLs represent promising therapeutic targets in breast cancer. Among current strategies, antibody-based agents (e.g., anti-GD2), S1P-related drugs (e.g., SK1-I, ABC294640, FTY720, NOX-S93, Sphingomab^®^), and ceramide-based delivery platforms exhibit the greatest potential. However, antibody therapies require further refinement to reduce off-target neurotoxicity linked to GD2 expression [154,155] and to enhance tumor specificity through advanced engineering or tumor-selective antigen variants. Modulators of S1P or S1PRs may also complement CAR-T cell therapy, although their widespread expression necessitates caution regarding systemic effects. Ceramide-integrated nanocarrier systems have demonstrated efficacy in overcoming drug resistance [142,148,150]; nonetheless, further optimization of nanocarrier systems, including particle size, surface characteristics, co-delivery strategies [165], and release kinetics, is critical for achieving precise biodistribution and targeted delivery. Personalized nanomedicine approaches, adapting ceramide species or carrier composition to distinct breast cancer subtypes, may further enhance therapeutic efficacy.

A key limitation remains the absence of potent, selective inhibitors of SL metabolism. Few isoform-selective CerS inhibitors have progressed beyond preclinical studies [166]. Moreover, while small-molecule inhibitors targeting SphK1, SphK2, and the S1P/S1PR signaling axis (e.g., ABC294640) have reached early clinical evaluation [167], none have achieved approval. Additionally, since most SL-metabolizing enzymes are intracellular [3], current antibody therapies are restricted to surface targets such as GD2 and S1PRs. Considering that the mitochondrial respiratory chain has recently emerged as a major therapeutic target in breast cancer [168], modulation of the ceramide pathway, which affects mitochondrial respiratory chain function [31–33], may represent a promising strategy for breast cancer therapy. Future strategies integrating SL-targeted therapeutics with immunotherapy or precision oncology approaches may offer a more comprehensive means of managing breast cancer. Such combinatorial regimens, supported by rigorous clinical validation and advanced formulation technologies, hold promise for achieving durable and selective antitumor responses with minimized systemic toxicity.

## 6 Conclusion

Breast cancer represents one of the most prevalent malignancies among women and encompasses a heterogeneous group of subtypes. Despite advances in therapeutic strategies, breast cancer treatment remains challenging due to the heterogeneity of its known subtypes, the complex tumor microenvironment, the emergence of drug resistance, and the high potential for metastasis. Breast cancer has been extensively investigated with regard to SL metabolism, revealing numerous potential molecular targets. This growing body of evidence underscores the therapeutic potential of SL-targeted agents, which may serve as effective monotherapies or can be integrated into combination regimens to enhance therapeutic efficacy.

## Data Availability

Not applicable.
